# Doctoral programmes in the nursing discipline: a scoping review

**DOI:** 10.1186/s12912-021-00753-6

**Published:** 2021-11-15

**Authors:** Beata Dobrowolska, Paweł Chruściel, Anna Pilewska-Kozak, Violetta Mianowana, Marta Monist, Alvisa Palese

**Affiliations:** 1 Department of Holistic Care and Management in Nursing, Faculty of Health Sciences, Medical University of Lublin, Staszica Str. 4-6, Lublin, Poland; 2 Department of Nursing Development, Faculty of Health Sciences, Medical University of Lublin, Staszica Str. 4-6, Lublin, Poland; 3 Department of Gynaecology and Gynaecological Endocrinology, Faculty of Health Sciences, Medical University of Lublin, Staszica Str. 4-6, Lublin, Poland; 4 2nd Department of Gynaecology, Medical University of Lublin, Jaczewskiego Str. 8, Lublin, Poland; 5 Department of Medical Sciences, University of Udine, Viale Ungheria, 20, 33100 Udine, Italy

**Keywords:** Doctorate Education, Doctorate of nursing practice, PhD/doctorate in nursing, Nursing Discipline, Research, Scoping review

## Abstract

**Background:**

This study aimed to map and summarise the state of the research regarding doctoral programs in nursing, as well as the issues debated in the context of nursing doctoral education. A Scoping Review in accordance with the Preferred Reporting Items for Systematic reviews and Meta-Analysis extension scoping reviews statement (PRISMA-ScR) was conducted. Three electronic bibliographic data bases were searched: Cumulative Index to Nursing and Allied Health Literature Complete, Medline (on EBSCO Host) and SCOPUS to identify empirical studies published between January 2009 and December 2019. The review process was based on framework identified by Arksey and O’Malley and further revised by Levac and colleagues. Analysis was performed with the use of the Donabedian framework regarding the structure of the doctorate programmes, the process, and the outcomes.

**Results:**

The review included 41 articles, mostly originating in the United States (*n*=26) and Europe (*n*=8), mainly by collecting the perceptions of students and faculty members with descriptive studies. The following issues were investigated at the (a) structure level: *Prerequisite for doctoral candidates*, *Qualifications of faculty members*, *Mission of doctoral programs*; (b) process level: *Doctoral programs contents, Doctoral programs resources and quality, Mentoring and supervision, Doing doctorate abroad*; and (c) outcome level: *Academic performance outcomes in doctoral programs, Doctoral graduates’ competences, Doctoral students/graduates’ satisfaction, Doctoral graduates’ challenges.*

**Conclusions:**

Doctoral programs have mainly been investigated to date with descriptive studies, suggesting more robust research investigating the effectiveness of strategies to prepare future scientists in the nursing discipline. Doctorates are different across countries, and there is no visible cooperation of scholars internationally; their structure and processes have been reported to be stable over the years, thus not following the research development in nursing, discipline and practice expectations. Moreover, no clear framework of outcomes in the short- and long-term have been established to date to measure the quality and effectiveness of doctorate education. National and global strategies might establish common structure, process and outcome frameworks, as well as promote robust studies that are capable of assessing the effectiveness of this field of education.

## Background

The doctoral education of nurses has been reported across the world to follow different traditions; as it was not previously possible to obtain a doctorate in the nursing discipline, nurses have been doctoral-educated in disciplines other than nursing [1]. In some countries, such as the United States (US), nurses have been allowed to obtain a doctorate in education since the early 20th century [1–3]; however, doctoral programmes were reported to become nursing-oriented in the 1970 s [4]. In other countries, for example Nordic ones, nursing doctoral programs started to operate a few decades later [5], while in others, for example Slovenia, this was just a couple of years ago, also as an effect of Bologna Process across Europe re-designing the educational cycles [6, 7]. There is no doubt that doctoral-prepared nurses performing research are crucial [8, 9] and they are required to have an effective scientific education [7, 10, 11].

In recent years, doctoral education in nursing has gained increased attention; a growing number of nurses have been reported to be engaged in doctoral studies [3, 5, 11, 12] due to the need for high quality clinical nursing practice, nursing education and science [13, 14]. Nevertheless, the trend of ageing of faculty nurses and their shortage has been debated for over 40 years [15–17] and different options have been discussed to increase the number of doctoral-educated nurses [15, 16, 18].

Moreover, different roles of doctoral-educated nurses have been documented in academia and in clinical settings [19] and challenges regarding competition in the ‘scientific market’ have been underlined, suggesting that the nursing discipline must be strengthened and recognised in high quality publications [9]. Therefore, while nurses with a doctorate are expected to be engaged in research projects [8], they are also expected to improve the quality of nursing care by changing the education and practice; thus, they are facing multiple expectations [19, 20]. As a consequence, nursing scholars and leaders are looking for options to develop doctoral studies into the most effective way.

In this context, many primary studies have been published to date (e.g. [10, 12, 19, 21]). However, the available studies have never been summarised in an accessible document that could inform future actions regarding the development of doctoral programmes. Therefore, summarising the state of the art of the research in this field, as well as the issues debated in the context of nursing doctoral education, are the main aims of this scoping review.

### An overview of doctoral education in nursing

There are many different nursing doctoral programs across the world, with different solutions regarding titles, the curriculum, competences and career possibilities. Even within a single country, these programs are different and, as highlighted by McKenna et al. [7], most of them have not included any cross-country collaboration regarding research lines and the curricula. In several countries, for example the US and the UK, two doctorate profiles have been established: the Doctor of Nursing practice (DNP), defined as a clinical or professional doctorate, and the Doctor of Philosophy (PhD), defined as a research doctorate. They have different aims regarding the discipline and practice development: a PhD is research-focused, whereas a DNP is focused on preparing future clinical leaders by guiding evidence-based nursing practice; in the US, it is required as an entry level for advanced nursing practice (ANP) [20, 22–26]. Discussions regarding what competences these programmes should ensure and what paradigm should be established when educating nurses on doctoral studies are still open [20]. Moreover, the development of collaborations between these two traditions of education has been underlined with the intent to promote the quality of care [27, 28].

Even though the number of doctoral-prepared nurses is increasing, difficulties in recruiting nurses to doctoral programs have been documented [29]. Firstly, nurses are more attracted to gaining clinical experience and becoming faculty members later; additionally, there are some barriers to entry into doctoral education, for example heavy nursing care workloads, high competition, and modest salaries. Moreover, other barriers are also set in the following stage, with regard to being recruited and remaining in the faculty: despite the great demand for nurses in the faculty, those who are already appointed have been reported to have high burnout and an intention to leave the position [30, 31], as well as due to the excessive pressure regarding publications, projects, and grants [17]. Additionally, while some countries have established the requirement that nursing departments must recruit staff with a research-doctorate (e.g., the US, Australia, China, South-East Asia), others, such as the UK, are still in continuing transition, employing nurses at the university level, without PhDs, and some are even employed without master’s level qualifications [32].

Different innovations have been discussed in order to prevent the lack of nursing scholars, such as establishing new pathways to obtain doctorates, allowing new graduates to access the doctorate programme directly after the BNS (Bachelor in Nursing Science) or a pre-baccalaureate to the PhD programme with individually tailored curricula [16, 18, 33]. Even though this option is criticised by some academics because of a lack of clinical experience before entry to doctoral programs [18], such candidates are young and may have a longer career as researchers, which is important when considering predictions regarding retirement trends among the faculty [18].

In addition to the above-mentioned factors, some researchers (e.g. Mckenna et al. [7]) also discussed the quality of doctoral programs. An urgent need to change these programs to support the advancement of nursing science has been stated [10]. Moreover, the need to enrich nursing doctorate education with knowledge of other disciplines, e.g., humanistic, social or biological sciences [34, 35], as well as in quantitative methods [8], have been solicited. Given that research findings must be published to inform developments of the nursing discipline, different methods are under discussion regarding the dissemination of doctoral dissertations [36] and increased popularity has been achieved by using the manuscript dissertation format [37].

The post-doctorate programme is also debated: McNelis et al. [12] reported that nursing doctoral students have not been prepared for the academic role, specifically for teaching; while Bullin [19] also added considered their competences in implementing innovative methods in education, suggesting that they require additional preparation though a revision of the curriculum [12]. Moreover, the roles of doctoral-prepared nurses in clinical settings are also discussed. Andreassen and Christensen [38] highlighted that those nurses holding a doctorate should change their practice, functioning as a leader in incorporating the evidence in the clinical field. However, experienced clinical nurses with doctorates have been documented to encounter several challenges when they start working in academia [17] suggesting that a clear career strategy should be developed for those willing to stay in a clinical setting.

## Methods

### Design

A scoping review has been performed by following the available frameworks [39, 40] in the following steps: (1) research question identification; (2) relevant studies identification; (3) studies selection; (4) data charting; and (5) results collation, summary and report. Specifically, methods and findings have been reported according to the Preferred Reporting Items for Systematic reviews and Meta-Analysis extension-Scoping Reviews (PRISMA-ScR) statement [41].

### Research questions

The following research questions were addressed: (a) What is the state of the research in the nursing field regarding the doctorate programmes, and (b) what are the main issues debated to date in the available literature?

### Studies identification

A comprehensive electronic database literature search was conducted in January 2020. The Boolean operator AND was used with combinations of search terms including the following: PhD/doctorate in nursing, competence and career pathways. The Cumulative Index to Nursing and Allied Health Literature (CINAHL) Complete, Medline (on EBSCO Host), and SCOPUS were searched to identify articles published between January 2009 and December 2019. This period was chosen considering two main reasons: (a) the availability of a previous review regarding doctoral nursing students’ persistence and the challenges faced by them, covering sources published between 1985 and 2011 [42], and (b) the Bologna Process regulating education cycles across Europe that has reached its 20 year anniversary in 2019: specifically, 2010 was the year which was established as the aim of European Higher Education Area development [6].

**Fig. 1:**
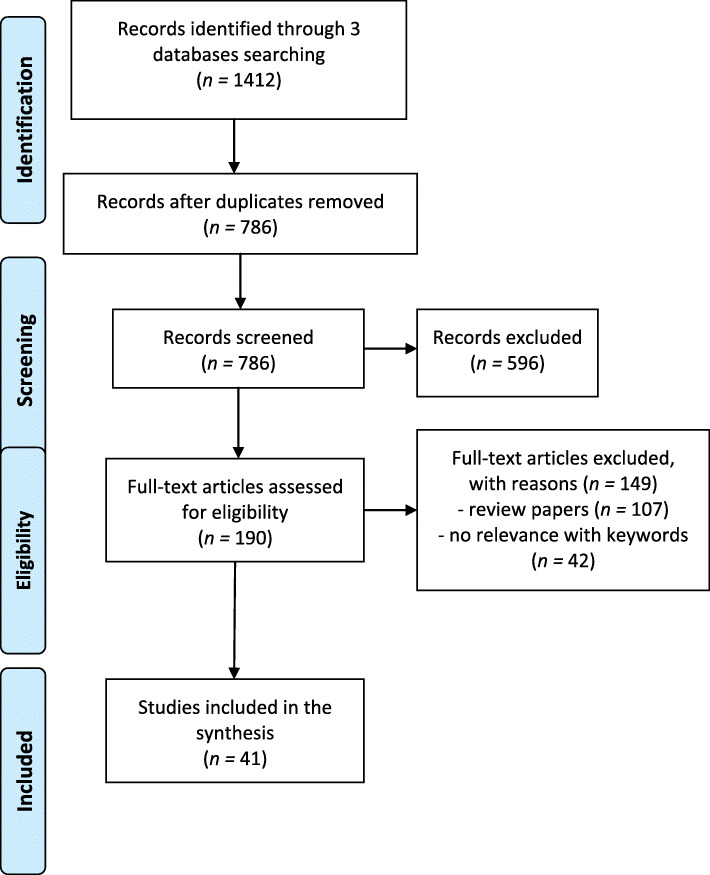
Flow diagram search and selection process of scoping review [41, 62].

Articles written in English, peer-reviewed, with an available abstract, and reporting both primary (qualitative, quantitative and mix-methods), and secondary (systematic reviews and meta-analysis) data were included. A total of 1412 records were identified; after screening and eligibility analysis, 41 articles were deemed eligible (Fig. 1). Therefore, articles not meeting these criteria and those focused only on problems and the situation of faculty members with a doctorate (e.g. [31]), were not included.

At the first level, titles and abstracts were screened by two researchers independently and then the findings were discussed. In the second phase, the eligible studies were evaluated through full texts in an independent fashion by two researchers; when these satisfied the inclusion criteria and the researchers agreed, the study was included. In cases of disagreement, other researchers engaged in the analysis were contacted, and when agreement was reached the study was included or excluded.

### Data charting

The following data expressing the characteristics of studies were extracted from each included study: (1) author(s); (2) publication year; (3) country; (4) study aim(s); (5) method(s) and research design; (6) participants (when available); and (7) main findings relevant to the aim of our study. The grid was piloted among five studies and then used for all included articles. Two authors independently assessed and extracted the data and agreed upon the findings. Discrepancies were discussed with other researchers.

### Results collation, summary and report

In line with the two-fold research questions, the included studies were considered analytically according to their main features: first, the main characteristics of the studies were summarised, and then, with regards to the second aim, issues were categorised according to the Donabedian [43] framework for issues regarding the structure of the doctorate programmes, the process, and the outcomes. Under the *structure* component, we categorised the prerequisite for doctoral candidates, their motivation, the qualifications of the faculty members, the organisation of the doctoral programs, and the mission of the programme. Under the *Process* component, we included doctoral programme implementation, strategies and methods used in education, and interactions between the faculty, the doctoral students, and other stakeholders. Under the *outcomes* component, the results of doctoral programs documented at different levels (e.g., doctoral graduates, nursing as a discipline, doctoral students) were categorised. In some cases, difficulties were found in categorisation, as overlap exists between the three main categories. Two researchers categorised the main findings according to the Donabedian model [43] and other researchers resolved inconsistencies. At each stage of the analysis, considering the Donabedian model, differences between doctoral programs, if any, were highlighted and reported.

## Results

### What is the state of the research in the nursing field regarding the doctorate programmes?

As reported in Table 1, the included articles mainly represent four continents: North America (*n*=27) [2, 3, 8, 10–13, 15–20, 27, 28, 30, 33, 36, 37, 44, 49–54]; Europe (*n*=8). [5, 7, 24, 25, 35, 38, 48, 57], Asia (*n*=4) [14, 46, 47, 55], and Australia [21]. Two are multi-country studies [5, 45], and most studies originated in the USA (*n*=26; [2, 3, 8, 10–13, 15–18, 20, 27, 28, 30, 33, 36, 37, 44, 49–54]). Most of the studies were published in the last 6 years (*n*=28; [3, 5, 8, 10–17, 19–21, 27, 28, 30, 33, 35–38, 45, 48, 54–57], with 11 in 2019 [3, 5, 11, 12, 17, 20, 27, 35, 36, 56, 57].

**Table 1:** Characteristics of the included studies

No	Author(s)/ year/ country	Aim(s) of the study	Method and research design	Sample	Relevant main findings
[2]	2012 (USA)	To determine the perceived feasibility of the eligibility for tenure being granted to nurses prepared at the level of doctor of nursing practice (DNP)	Internet- based survey	Faculties and deans (*n*=65) from a randomly chosen list of doctor of nursing programs from the American Association of Colleges of Nursing	In 61.3 % of the institutions, DNP faculty members were eligible for tenure. 56.25 % of respondents reported that their institutions considered practice in granting tenure. According to 75.4 % respondents allowing DNP faculty eligibility for tenure brings benefits, such as recruitment and retention of faculty, parity with other practice doctorates, and the clinical component that they bring. 41.27 % respondents reported that they have concerns related to allowing DNP faculty eligibility for tenure, such as the fact they are not adequately trained in the research process, and that DNP tenure diminishes the progress nursing has made in academia.
[3]	2019 (USA)	To evaluate mentoring of doctoral students’ work by nursing faculty in DNP and PhD programs	Descriptive study with online survey	Nursing faculty members (*n*=230; (DNP=177 and PhD=53).	Mentoring is crucial in effective doctoral education. A number of students were identified for effective mentoring (PhD M=3.4, DNP M=4.3). Specific qualification to mentor doctoral students of both programs is required, however only ¼ faculty members received formal training in this regard. Among the factors that would facilitate effective mentoring by faculty are allocation of time, number of students, students’ readiness for doctoral work, better preparation for the mentoring role, and resources which support students’ work.
[5]	2019 (Nordic countries)	To investigate and compare the prevalence of PhD-prepared nurses employed at university hospitals in the Nordic countries, to investigate what functions they fulfil, what research activities they undertake and how they describe their ideal work life	A descriptive cross-sectional study with online questionnaire	PhD-prepared nurses working at a university hospital in one of six Nordic countries (*n*=166)	The following functions of PhD-prepared nurses working at university hospitals were reported most often: (a) research, (b) teaching, (c) supervision, (d) administration. Only part of their time is spent on research. The majority of respondents held a position shared between hospital and university.
[7]	2014 (UK)	To evaluate the quality of doctoral education in nursing in the United Kingdom	A quantitative study with a cross-sectional comparative survey	Doctoral students/graduates (*n=*97) and faculty (*n=*37)	The results provide information regarding the program, faculty/staff, resources and evaluation. Staff members were more likely to agree with the statement: ‘your institution values, supports and provides rewards to students for research and scholarly activity’ than students. Staff members were more likely to agree that the emphasis of the doctoral curriculum is consistent with the mission of the university and the discipline of nursing. Staff members think that students have ethical training within the framework of preparation for undertaking research. Students do not agree with this. Students disagree significantly more often with the statement ‘staff members provide students with diverse and challenging learning experiences’ than staff members. Most students reported that the environment and resources, including financial support and the time allocated to support students, were of poor quality. Students rated supervision as excellent. Students rated the overall quality of the program higher than staff members.
[8]	2018 (USA)	To describe development of new competency-based curriculum in quantitative research for PhD nursing students	Mix-method study: assessment of quantitative research methods curricula; survey of PhD students and alumni; interview with program faculty	PhD students (*n*=127*);* top National Institute of Health-funded nursing schools with PhD degree programs (*n*=8)	The study revealed that students are interested in practising with secondary data analysis using large data sets, biomedical informatics data interpretation, an understanding of applied machine learning algorithms, and to improve their understanding of complex database management. Students requested more in-depth statistical courses. All surveyed programs offered a year-long training course in epidemiology and quantitative research methods (some schools offered 2 years of training).
[10]	2015 (USA)	To examine the content of U.S. PhD programs in nursing as communicated on the program websites in 2012	A descriptive design using Web scraping methodology was used	Research-focused doctoral (PhD and DNS) programs in nursing (*n*=120) in USA	Nearly all programs included statistics/quantitative design, philosophy/theory development and qualitative methods. Only 55 % of programs showed evidence of including nursing inquiry, and 43.3 % included research ethics. Education/teaching was included in 55.8 % of programs, policy in 50 % and leadership in 36.7 %.
[11]	2019 (USA)	To assess characteristics and practices of nursing PhD students, the mentoring practices of their advisors, and the likelihood of self-reported career readiness	A nationwide descriptive, cross-sectional study	PhD students (*n*=380) representing 64 schools	The mean self-rated scientific proficiency score of PhD students was 87.9 ± 13.4 (range: 20–120). A high percentage of students had positive reports of mentorship characteristics of their advisors. Greater readiness for their career was found among students who are older, with a greater number of responsibilities, and who work more hours per week. Mentoring practices were not found to be significant predictors of career readiness. However, having one or more mentor and advisor influenced greater readiness for career.
[12]	2019 (USA)	Understanding doctoral nursing students’ and recent graduates’ expectations of their educational experience related to preparation for an academic career	Descriptive qualitative design	PhD and DNP students and recent graduates (*n*=24)	Two major themes emerged in the analysis: (1) met and unmet expectations of programs; and (2) equivocal preparation for teaching.
[13]	2015 (USA)	To profile the nursing faculty in the United States teaching in PhD and DNP programs	A descriptive study with an online survey	Nursing faculty (*n*=554) who teach in PhD and DNP programs in the United States	Faculty who teach only DNP students are more likely to hold a DNP degree, while those teaching only in PhD programs or in both programs are more likely to hold a PhD. Faculty teaching only in DNP programs are more likely to engage in clinical practice. Faculty teaching in PhD programs only or in both programs were more likely to serve as members of doctoral students’ dissertation or project committees, provide academic counselling, mentor students in research, serve as committee chairs, provide informal mentoring to doctoral students, and supervise postdoctoral fellows.
[14]	2018 (China)	To investigate career intentions of nursing PhD students	Online cross-sectional survey	PhD nursing students (*n*=89)	For most of the PhD students, pursuing a PhD was part of their career plan (73.0 %). Regarding career intentions, most of the students (60.7 %) wanted to work in an educational institution. The most often indicated fields of work after graduation were nursing education (75.3 %) and nursing research (70.8 %), only a few students wanted to work in a clinical setting (16.9 %). Students’ expectations regarding the work role were: - the opportunity to put their strengths to the fullest use (79.8 %), - time to conduct research (60.7 %) - regular office hours for good work-life balance (51.7 %).
[15]	2016 (USA)	To describe the factors influencing the pursuit and completion of doctoral education by nurses intending to seek or retain faculty roles	The quantitative survey	Nurses, current students or recent graduates of doctoral programs (*n*=548)	Time was an important factor considered when choosing a doctoral program, both by PhD and DNP students/graduates. Time for degree completion was more important for DNP then for PhD students (the sooner the better). Almost all respondents reported that family and job obligations interfered with studying and writing. Nearly all respondents stated that their doctoral education was worth the time commitment. Money was another important factor considered when deciding to enter a doctoral program. Three aspects were crucial here: (a) paying for education, (b) return of investment in the future, and (c) impact on their future salary. Majority of respondents reported that doctoral education was worth money investment.
[16]	2016 (USA)	An evaluation comparing the early-entry option with two more conventional entry points was conducted	Qualitative and quantitative study	Three groups (*n=*84): (a) early-entry students, undergraduates or immediately upon graduation (*n=*29), (b) mid-entry students with baccalaureate degrees and at least 1 year of work experience (*n=*27), (c) delayed-entry students with master’s degrees and 1 or more years of work experience (*n=*28)	Similarities and differences emerged between students who enter a PhD program in nursing before their baccalaureate degree and students after their baccalaureate degree and after their master’s degree. In qualitative data, among the similarities, three themes emerged: availability of funding, mentors and teaching preparation. Among the differences, two themes emerged: Career decision-making and clinical competence. In quantitative data, among the similarities, research productivity and faculty position in nursing as post-graduation employment were reported. Among the differences: diversity regarding age and ethnicity of students and progression measures were identified.
[17]	2019 (USA)	To explore the experiences of DNP and PhD prepared faculty on tenure-track in academia through narrative stories	A qualitative narrative design	Doctoral-prepared nurses (*n*=19) on tenure-track.	Five themes were found: (1) The ability to develop meaningful partnerships, (2) A necessity to balance responsibilities, (3) Destructive criticism is real, (4) I have value in academia, (5) Multifaceted coaching to produce achievement.
[18]	2014 (USA)	To (1) evaluate admission criteria into PhD programs for direct entry from a bachelor’s degree; (2) ascertain bachelors and masters’ degree nursing students’ perspectives on pursuing a BSN to PhD; (3) clarify factors that influence students’ decision-making processes behind pursuing a PhD	A cross-sectional pilot study	Currently enrolled bachelor’s, master’s, and doctor of nursing practice students (*n*=606)	Chosen admission criteria into PhD programs from a bachelor’s degree: 5 programs required at least one year of clinical work experience as RN. Other are also: a graduate level statistics course, admissions interview, and grade point averages (GPAs) – which is set on different levels for different programs. 69 % of the surveyed students indicated that they may or definitely would consider a PhD directly after their current program of study. Among the barriers for pursuing PhD study, three emerged in qualitative analysis: cost, time and experience. Among the students’ areas of interest for PhD study, the most often indicated was non-communicable diseases, followed by global health.
[19]	2018 (Canada)	To understand the state of the literature regarding a PhD requirement and the extent to which a PhD supports academic nurse educators in their teaching roles	Integrative review	Peer-reviewed papers *n*=139	The majority of published works originated in the USA (*n*=126). Only 33 studies were research-based. Results are framed in following themes: (a) What is an effective educator? (b) What is the current practice for the formal preparation of teachers in higher education? (c) How is excellence in teaching described? (d) What conditions influence or have an impact on academic nurse educator preparation for the responsibilities of their roles?
[20]	2019 (USA)	To develop a substantive theory about the perceptions and the attitudes of doctoral nurses regarding their roles	An adapted approach of Strauss and Corbin’s grounded theory methodology. The semi-structured individual interview and focus group interviews	Doctoral-prepared nurses (*n*=*13)* and nursing experts (*n*=5) with expertise regarding doctoral studies	The core category that emerged was *Following the Path* – describing respondents’ perspective of the PhD and DNP roles. Additionally, four categories were identified: (1) advancing (perceiving the doctoral role, moving practice forward, influencing); (2) collaborating (working together, building the identity, identifying); (3) transforming (complex health care, changing, choosing roles); and (4) stewarding (building the profession, performing the role, mentoring/growing)
[21]	2018 (Australia)	To understand the experiences of nurses and midwives enrolled in a PhD, explore barriers that PhD students encounter whilst completing the degree, and develop recommendations for support strategies to encourage completion a PhD degree	A mixed methodology, non-experimental design approach	Registered Nurses and Registered Midwives enrolled to PhD program (*n*=16)	Results regard the following aspects of PhD programs: supervision, feedback, peer support groups, supervisors: expectations/viewpoints, and the PhD journey. Most PhD students give positive comments regarding their supervisors. Feedback received from supervisors regarding submitted work was usually timing, not always consistent, often including conflicting advice, sometimes unhelpful and difficult to understand. Peer group support was not highly rated by the students. More than half of the surveyed students reported that they understand their supervisors’ expectations and viewpoints. Half of the PhD students thought the PhD had been a positive learning curve.
[22]	2011 (UK)	Reports on a national study that sought to investigate the learning expectations and experiences of overseas doctoral nursing students in the UK.	Semi-structured qualitative interviews	International doctoral nursing students (*n*=17) representing 9 different countries from 6 different UK universities	Three main categories emerged from the analysis: (1) A journey of transitions: adjusting to doctoral study in the UK (themes identified: expectations and reality, anxiety and challenge: adjusting to UK academic practices, learning in another language) (2) A journey of relationships: finding support for doctoral study (themes identified: negotiating the complexities of supervision, peer support, institutional support) (3) A journey of challenge and a journey of growth (themes identified: an emotional journey, transformation).
[23]	2009 (UK)	To explore student and supervisor perceptions and experiences of the research supervision process within a professional doctorate programme.	An exploratory, descriptive approach	Students (*n*=15), and professional doctorate research supervisors (*n*=5). Additionally, convenience sample of students (*n*=2) and supervisors *(n*=2) for one-to-one discussion.	Seven following themes emerged from the analysis: (a) Supervisor style; (b) Pragmatism; (c) Broken discourse: independence; (d) Broken discourse: facilitation; (e) Partnership and equality; (f) Posturing; (g) Professional issues
[25]	2019 (USA)	To describe DNP and PhD collaboration in an academic setting	Two examples analysis	Collaborators faculty and collaborators students *(n*=4)	PhD and DNP faculty members can utilise complementary skill sets in order to prepare projects which are scientifically sound and practically important. Two programs of faculty collaboration may be extended on PhD and DNP students’ collaboration.
[26]	2017 (USA)	To explore attitudes and determinants for effective collaboration among doctoral-prepared nursing faculty	Qualitative study	Four focus groups included faculty members (*n*=41) who taught in DNP and/or PhD programs.	Five themes emerged: (a) DNP not well understood, (b) Confusion surrounding research, (c) Opportunities for collaboration, (d) Lack of structural support, (e) Personal characteristics and attitudes.
[28]	2016 (USA)	To identify barriers and facilitators to academic careers for doctoral (PhD) nursing students	Cross-sectional study	PhD students (*n*=933)	72.5 % of respondents planned academic careers, and they were more likely to work in teaching or research (71 % vs. 15 %). The average age of students entering doctoral program is 33.9 – for post-baccalaureate level, and 43.4 at the post-master’s level. The first group would graduate in 5.2 years and the second in 5 years. Participating in teaching development activities, receiving financial support and having a faculty member mentor during doctoral education influence students plans regarding future academic careers. 47 % of students who had non-academic career plans switched to academic career plans during their doctoral study.
[31]	2018 (USA)	To explore the unique characteristics of the direct entry BSN/BS-PhD student experience	Descriptive qualitative design. The modified Delphi method was used.	Panel experts (*n*=4) (current students or recent graduates of BSN/BS-PhD programs).	Four themes were identified as BSN/BS-PhD students experience: (1) Commitment to science, (2) Nursing identity, (3) Exploring prospects, (4) Balancing family and student expectations.
[33]	2019 (UK)	To examine how PhD theses in nursing may be categorized, what they study, what theoretical approaches they employ and, to what degree nursing theory is employed as a current theoretical approach.	Descriptive qualitative design	PhD theses (*n*=61) in nursing science published from 1994–2015, at the University of Edinburgh	Analysis shows that only a few of the PhD theses referred to nursing theory and few used it as their theoretical approach, or as part of the theoretical approach. The vast majority of the theses referred to theories developed by disciplines other than nursing.
[34]	2019 (USA)	To compare dissemination of PhD dissertation research by dissertation format	Retrospective study (1999-2019) – analysis of dissertations (traditional format) and publication dissertation – in PubMed	PhD graduates (*n*=113)	The majority of PhD graduates employed traditional format for their PhD dissertation (70.8 %). 41.3 % of them had never published dissertation findings in peer-reviewed journal. Those graduates who chose an alternative format for their dissertation, had a higher number of peer-reviewed publications.
[35]	2018 (USA)	To explore the advantages and disadvantages of the traditional format vs. manuscript option for dissertations among nursing PhD programs in the United States	Cross-sectional census survey	PhD programs in USA (*n*=79)	Among the programs surveyed, 84 % offered the traditional format and 71 % offered the manuscript option format. The majority of programs (59 %) offered both formats. Among the reasons why programs adopted the manuscript option dissertation format, two were most often indicated: the PhD program faculty supports this kind of dissertation and it may lead to an increase in the transition of student nurse scholars to academic positions. Among the advantages of the manuscript option dissertation format, future career opportunities for students (academic position) were highlighted. Among the disadvantages: challenges with formatting and a lack of writing skills. From programs offering the manuscript format, the majority (61 %) required three manuscripts in order to graduate.
[36]	2018 (Denmark)	To explore different perspectives on the positioning of PhD nurses and how they contribute to clinical nursing practice	A qualitative, explorative interview study	PhD nurses (*n*=6), nurse colleagues (*n*=9) and clinical nurse leaders (*n*=6)	Nurses with a doctorate see themselves as those who change clinical practice to evidenced-based. Also, their colleagues expect that they will implement research results into practice. Nurses with a doctorate are perceived as important resource, so they should raise the standard of clinical practice by doing useful research. According to study findings, the position of PhD nurses in clinical setting is uncertain and unstable.
[42]	2011 (USA)	To investigate the number of clinical hours required in postmaster’s programs and the types of clinical experiences provided	Prospective, descriptive cross-sectional study	DNP program directors (*n*=43)	The number of required clinical hours ranged between 0 and 1,000. None of the schools required additional practice hours solely to supplement previous master’s-level supervised clinical hours. However, 20 % of schools reported having a separate clinical course, 26 % of schools’ clinical hours have end-of-program practice immersion experiences, 38 % used both as supplemental and for end-of-program immersion experiences. Supervision of clinical hours: 45 % responded that they are supervised by DNP faculty, 19 % reported that they are completed independently, 57 % are mentored by a preceptor in the clinical setting.
[43]	2015 (7 countries)	To compare the findings of the quality of nursing doctoral education survey across seven countries and discuss the strategic directions for improving quality	A descriptive, cross-country, comparative design with an online questionnaire	Deans/schools (*n*=105), faculty (*n*=414) and students/graduates (*n*=1149) from nursing schools in seven countries: Australia, Japan, Korea, South Africa, Thailand, UK and USA	Both faculty and students/graduates rated the overall quality of nursing doctoral education as good to excellent. Among the four domains assessed in the survey, the highest average score was for the faculty domain, followed by the programme, evaluation and resource domains. Faculty assessed the quality higher than students/graduates in three domains (programme, faculty and evaluation).
[44]	2010 (South Korea)	To describe the perceived quality of Korean nursing doctoral education in faculty, students, curriculum and resources	A qualitative research design (focus groups)	Four groups: deans (*n*=10), faculty (*n*=7), students (*n*=7) and graduates (*n*=6).	Themes emerged with regard to strengths and weaknesses of Korean nursing doctoral education. Among the strengths of the faculty are e.g. recognition of the faculty’s research productivity, ability of the faculty to attract extramural funding, and new research methods delivered by young faculty. Among the weaknesses of the faculty are e.g. teaching courses without content expertise, ageing of the faculty and insufficient faculty with expert knowledge in nursing. Among the strengths of the students are e.g. students with diverse educational and institutional backgrounds and flexible university policy regarding admission. Among the weaknesses of the students are e.g. declined quality of students and decreased number of doctoral applicants. One of the strengths of the curriculum is interdisciplinary courses. One of the weaknesses of the curriculum is a lack of courses to develop core research competencies. Among the strengths of the resources there is e.g. inter-institutional courses with credit transfer. Among the weaknesses of resources there is e.g. a lack of funding support for research.
[45]	2011 (Jordan)	To retrospectively explore: (a) how individuals experienced the doctorate; (b) what they felt they had learned from it; (c) which factors influenced the further development of research activity	Qualitative study	PhD nursing graduates in Jordan who had studied in the UK (*n=*16)	The following themes emerged from the study: (a) Difficult discovery journey; (b) Transformation of oneself; (c) Passing it on.
[46]	2016 (Denmark)	To create awareness among nurse leaders of what they may need to consider when integrating nurse researchers as advanced nurse practitioners (ANP) at PhD level among their staff	A collective case study	ANPs with PhD (*n=*3) at a large regional hospital in Denmark	Abilities of ANPs with a PhD that emerged from the study: the use of knowledge in practice, clinical thinking and analytical skills, clinical judgment and decision-making skills, professional leadership and clinical inquiry, coaching and mentoring skills, research skills and changing practice. All of these abilities are integrated with the implementation of evidence-based practice.
[47]	2011 (USA)	To identify current admission criteria and academic performance outcomes in nursing PhD programs	Descriptive exploratory design	Nursing PhD programs (*n* =56).	100 % of programs reported that Graduate grade point average (GPA) is considered in the admission to a PhD in nursing. The most often indicated minimum grade was 3.0. For 91 % of institutions, one or more examples of writing were required as part of the admission process. The most common type was a statement of goals (79 %) and a scholarly project (36 %). 98 % of institutions required a letter of recommendation. Applicant interviews with the faculty were required by 82 % of institutions. 91 % of participants indicated that research match with faculty was considered in the admission process. 6 common academic performance outcomes in nursing PhD programs were identified: comprehensive examination (80 %), ongoing minimum graduate GPA (79 %) of 3.0 (82 %), and a formal dissertation: chapter format (77 %), time to degree attainment (71 %), degree attainment (71 %), and time to candidacy (63 %).
[48]	2013 (USA)	(1) to describe key aspects of DNP program capacity, and (2) describe the potential impact of the DNP on faculty resources in nursing research doctoral programs	A survey	The deans of nursing schools (*n*=126) offering DNP programs	The average length of time taken for students to finish the MSN-DNP program was reported to be 2.43 years, whilst the BSN-DNP took 3.8 years. 81 % of programs reported the required research course. 79 % of programs required clinical practice in the program, more so in BSN-DNP than in MSN-DNP programs. Also, only 5 % required teaching practice. In 84 institutions, 232 faculty members who had been principal investigator of at least one research grant were employed in the DNP program. Among the 33 reporting institutions which have PhD and DNP degrees, only one reported no faculty overlap.
[49]	2013 (USA)	To uncover the lived experience of developing as a scholarly writer	Qualitative – hermeneutic phenomenology	Students enrolled in their first semester of coursework in a PhD in nursing (*n*=10).	Themes uncovered: (a) Coming to know about scholarly writing, (b) Shifting thinking in order to write scholarly, (c) Giving birth: the pain and the pleasure of scholarly writing, (d) Putting all of the pieces together into the final product.
[50]	2012 (USA)	To describe the resources available for research support in schools of nursing with doctoral degree-granting programs	Descriptive survey design, the online survey	The deans of nursing schools *(n*=120) offering doctoral degrees.	75 % (from 116 programs) reported having a research office within their institution, and 76 % of these schools provided information about their budget. The average budget for the research office in years 2008-2009 was $350,000. Among the major goals of the research office, the following were reported: to increase of the amount of external funding obtain (92 %), promote scholarly work including publications (90.8 %), and promote collaborative research with other disciplines (88.5 %). Among the most often indicated activities of the research office were: grant development (100 %), grant assembly (92.9 %), grant budget development (90 %), research seminars (90 %), and statistical consultation (84.3 %). With regard to the personnel of the research office, 97.1 % employed a research office director/dean, 78.3 % a grant administrator and 74.3 % statisticians.
[51]	2012 (USA)	To examine trends in the process, timing, and methodology of comprehensive and qualifying examinations in nursing doctoral programs in the United States	Exploratory, descriptive cross-sectional Study with online survey	Administrators from research-focused doctoral programs (*n*=45) from 27 states across the country	According to 47 % of respondents, the most common method of doctoral comprehensive/qualifying examination was a written take-home test, with 2/3 reporting subsequent oral examination. 24 % of respondents reported using a form of the traditional written, on-site examination, with few follow-ups involving an oral defence. 20 % of programs implemented requirement for a written publishable paper with follow-up oral defence of the paper. As 67 % of programs reported, the examination was developed by a PhD program committee or special examination task force.
[52]	2018 (USA)	To examine how the effects of environmental stressors predict the students’ intent to leave their current program of doctoral study	A descriptive survey design	PhD and DNP students (*n*=835)	Two types of stress were identified that significantly predicted students’ intention to leave: (1) Stressors related to program issues, primarily relationships between the student and the faculty/advisor. When program stressors rise also the intent to leave rises. (2) Stressors related to support issues, specifically from the family/friends. When family support declines, intent to leave rises.
[53]	2017 (Iran)	To explore the challenges of the acceptance of the role of a clinical educator by PhD-graduated nurses who are faculty members.	Qualitative exploratory study with semi-structured, face to face interview	Sample (*n=*13): 8 PhD graduates in nursing, 3 heads of departments of nursing, 1 educational vice chancellor of a nursing school, and 1 nurse	One main theme emerged in the analysis: “Identity threat” with 5 categories: (a) expectations beyond ability, (b) lack of staff’s relies on a PhD graduate’s performance, (c) poor clinical competencies, (d) doubtfulness, (e) obligation
[54]	2019 (USA)	To identify common experiences voiced by the DNPs who have returned to school for the second nursing doctoral degree.	Qualitative research study using a heuristic, descriptive phenomenological approach	DNPs (*n*=12) who have returned to school in pursuit of a PhD in nursing	Three themes were uncovered from the analysis: (1) wanting to know something more; (2) social-individual tension; (3) challenges faced to transformational learning.
[55]	2019 [55] (Sweden)	To investigate what registered nurses (RNs) with a PhD working in a clinical practice experience in terms of their role, function and work context.	Qualitative design, with semi-structured interviews	Registered nurses (RNs) with a PhD (*n=*13)	One concluding theme was formed: “Having the competence and desire to improve clinical nursing, but facing barriers”, and 19 sub-categories grouped into 4 categories: (a) striving to develop nursing care, with or without support; (b) being present in clinical nursing care as an intentional strategy; (c) contributing to the development of evidence-based nursing (EBN); (d) supporting and enabling nursing education and competence development.

The majority of studies used a quantitative approach (*n*=20; [2, 3, 5, 7, 10, 11, 13–15, 18, 30, 35, 37, 44, 45, 49, 50, 52–54]), while others applied a qualitative (*n*=15; [12, 17, 20, 24, 25, 28, 33, 35, 38, 46, 47, 51, 55–57]) or mix-methods design (*n*=3; [8, 16, 21]). Only one integrative review emerged [19] along with two case analyses (*n*=2; [27, 48]).

Most of the studies referred to PhD programmes (*n*=22) [5, 8, 10, 11, 14, 16, 18, 19, 21, 30, 33, 35–38, 47–49, 51, 53, 55, 57], with DNP analysed in 5 cases [2, 25, 44, 50, 56], while both programs were tackled in nine studies [3, 12, 13, 15, 17, 20, 27, 28, 54]; in addition, some articles have shown research regarding doctoral programs without specifying its kind (*n*=5) [7, 24, 45, 46, 52]. The majority (*n*=35) of studies investigated the experience and expectations of students/faculty members/directors of doctoral programmes and deans, ANPs with PhDs by involving between three [48] and 1,668 participants [45], with a total of 7,159 participants in all 35 studies. Of the remaining, three studies have analysed doctoral programmes (with the number of programmes analysed from 56 to 120, e.g. [10, 37, 49], the thesis produced (*n*=61, [35]), examples of collaboration (*n*=4, [27]) and studies published regarding PhD requirements (*n*=139, [19])).

### What are the main issues debated in the available literature to date?

#### Structure level: (1) Prerequisite for doctoral candidates

Two main issues emerged: regarding the admission criteria of doctoral programs and the doctoral candidates’ criteria/motivations for choosing the programme. Admission criteria for doctoral candidates have been documented as different: for example, Squires et al. [18], in the case of direct entry PhD programs from a bachelor’s degree, reported a requirement of at least one year of clinical experience as a registered nurse. The number of required clinical hours before the admission to the DNP has been documented to range between 0 and 1,000 [44]. Specifically, DNP programs do not require additional practice hours for supplementing previous master’s-level supervised clinical hours. However, 20 % of programs reported having a separate clinical course, 26 % reported an end-of-programme practice immersion experience, and 38 % required both a supplemental and end-of-programme immersion experience [44].

In the study conducted by Squires et al. [18] for direct entry PhD programs from a bachelor’s degree, a graduate level statistics course, an admission interview, and the Grade Point Averages (GPA) were also reported. Megginson [49] investigated the admission criteria in PhD nursing programs and documented that the GPA was required in 100 % of cases, set-up mostly at 3.0 as a minimum; moreover, 82 % of analysed US programs also considered the Graduate Record Examinations (GRE) scores during the admission decision. Furthermore, one or more examples of writing (e.g., the scholarly project), a letter of recommendation (by a Professor), the applicants’ interviews and the research line matching that of the faculty have been also reported.

On the side of the candidates, the decision to enter a doctoral programme (both PhD and DNP) has been underlined as being affected by financial aspects and funding availability [15, 16]. Specifically, three aspects have been reported as crucial [15]: (a) paying for education, (b) returning investments in the future, and (c) impacting future salary. When choosing the programme, respondents have been documented to consider what would best fit to their busy life [e.g., a hybrid form of education with online courses], but also the time available and that requested [e.g., for degree completion – which has been reported to be more important in the case of DNP than PhD]. Time has been considered as one of the three main barriers identified for bachelor/master/DNP students to start a PhD [18]. The other two barriers are costs and the issue of experience, as students want to gain clinical experience before entering the programme. However, 69 % of students sampled indicated that they would consider a PhD directly after their current programme of study [18].

Choosing a professional doctorate or not has been reported to be influenced by the information available to the potential candidates; students chose a PhD as they were not aware of the DNP [24]. According to a study performed in China [14], the majority of PhD students have been reported to pursue a doctorate according to their career plan (73 %) and to improve their research abilities (53.9 %). Science passion and motivation to work for nursing discipline development, and through science for the improvement of quality of life, was also reported among BNS/BS–PhD students. However, their challenge was the lack of experience in clinical practice. This has been reported as the nursing identity threat mostly due to the opinions of “older” nurses, and also challenged their future career prospects in case they were not productive as scientists. Moreover, their life is challenged because they are the youngest students in doctoral programs and are also dealing with financial and family responsibilities [33].

#### Structure level: (2) Qualifications of faculty members

The profile of faculty teaching in DNP and PhD programs has been documented as different [13]. Those staff who teach DNP students are more likely to have DNP degrees and be engaged in clinical practice, whereas lecturers with a PhD usually teach PhD students and are more likely to be engaged in research activities with students while mentoring research and supervising doctoral candidates or postdoctoral fellows. However, in a study among the deans of those nursing schools offering DNPs, only one of the 33 institutions with previous PhD and DNP students reported no faculty overlap [50].

#### Structure level: (3) Mission of doctoral programs

McKenna et al. [7] documented that staff members are more likely than students to agree that the emphasis of the doctoral curriculum should be consistent with the mission of the university and the discipline of nursing. Considering the growing popularity of DNP programs, e.g., in the US, divisions between PhD and DNP have been documented to be at need of discussion regarding further development and the aim of these programs [20]. Staffileno et al. [28] reported that DNP and PhD students have difficulties cooperating due to different ‘languages’ and the challenges involved in understanding the role of this new degree. However, the importance of promoting collaboration between PhD and DNP students has been underlined, and it is often an issue associated with the personal characteristics of individuals involved rather than an issue related to the type of degree. Specifically, PhD and DNP students can collaborate within the course and as graduates, e.g., DNP students have a clinical perspective, so they know the correct research question to ask, whereas PhD students have methodological knowledge and know how to ask questions. Moreover, PhD students/graduates may help DNPs to get financial support and grants when they experience difficulties, and they can mentor DNP in their scientific work [28]. In the context of cooperation between academia and clinical practice, Cygan and Reed [27] have provided an example where academia nurses and clinical nurses shared the complementary skills that they have in order to prepare projects to be scientifically and practically relevant.

#### Process level (1) Doctoral programs contents

There is a plethora of different subjects in nursing doctoral programs: in a US study regarding research-focused doctoral programs, all of those under investigation included statistics/quantitative design, philosophy/theory development and qualitative methods. However, only 55 % of programs showed evidence of including a nursing inquiry [10]. According to Minnick et al. [50], around 81 % of DNP programs in the US have been reported to require research courses, 79 % require clinical practice, with more BNS-DNP than MSN (Master’s degree)-DNP programs, and only 5 % require teaching practice. Regarding other issues related to doctoral student qualifications, 43.3 % of programs included research ethics, 55.8 % education/teaching, 50 % policy and 36.7 % leadership contents. A little difference between programs from 30 years ago, and those available at the time of the study have emerged, also suggesting that programs do not respond to research priorities [10].

Students have been reported to assess doctoral programs different to the faculty. In McKenna et al. [7], staff members reported that each student had relevant ethical training in preparation for undertaking research, and that they had provided students with diverse and challenging learning experiences (e.g., social, ethical, cultural, economic and political issues related to nursing, health care and research). On their part, students have reported disagreement with such statements.

Nursing PhD students have been reported to need more practice secondary data for analysis using large data sets, biomedical informatics data interpretation, and an understanding of applied machine learning algorithms. They also required more in-depth statistical courses [8]. Additionally, PhD students have highlighted that learning scholarly writing is more effective when you have someone to explain it (expert-faculty to guide) and when the support system in this scope is established [51].

#### Process level (2) Doctoral programs resources and quality

Having research support for students has been highlighted as important for doctoral education. In a US study, among the deans of nursing schools offering a doctoral degree, 75 % have reported offering a research office to increase the amount of external funding, to promote scholarly work including publications, and collaboration with other disciplines. In this line, grant development, assembly, budget development, research seminars and statistical consultation were the most activities performed by the office [52].

However, the resources and quality of the doctoral programmes are not always perceived homogeneously from the side of the faculty and the students. In a study involving seven countries, both faculty and students/graduates have rated the overall quality of nursing doctoral education as good to excellent. The highest average score was reported for the faculty domain. In all countries surveyed, the faculty assessed the quality higher than students/graduates in three out of four domains (namely: programme, faculty and evaluation) [45]. In contrast, in a single country study involving UK doctoral students and faculty, students assessed the quality of doctoral programs higher than faculty members. However, students did not agree that the environment and resources available, such as financial support, time allocated by staff to support students, and level of the technical support, were of an appropriate quality [7].

Strengths and weaknesses in the quality of doctorate programmes have been documented in a Korean study where the strengths included faculty research productivity, the application of new research methods, students’ diverse backgrounds and interdisciplinary courses. Among the weaknesses were the aging faculty, decreased number of candidates for doctoral study, and a lack of funding support for research [46].

In addition to the above-mentioned aspects of quality, doctoral programme quality indicators have also been reported in the doctoral comprehensive/qualifying examinations. Mawn and Goldberg [53] investigated research-focused doctoral programs, and reported different methods for such examination, ranging from the written take-home test to a written publishable paper with follow-up oral defence of the paper.

#### Process level (3) Mentoring and supervision

According to some authors [3, 16, 21], mentoring is very important in directing research during a doctoral programme (both PhD and DNP). Mentoring relationships have mostly been focused on the pursuit of scientific inquiry, the transfer of knowledge, facilitating research activities and developing research partnerships [19]. However, there are different expectations when mentoring doctoral students according to the degree programme. In the case of PhD students, faculty mentors are expected to hold a PhD, be engaged in research, publish articles, and have an overall scientific portfolio. In the case of DNP students, faculty mentors must hold a doctoral degree (DNP or PhD), be active clinical practitioners (e.g., ANPs) and have experience as a mentor in specific topics such as quality improvement and patient safety [3]. However, in a UK study, it was found that supervisors did not distinguish between the needs of professional doctorates and PhD students and reported the same expectations regarding research [25].

To be effective, mentors of doctoral students should be trained; also, the number of students allocated to a single mentor is important, as is the time available and the students’ readiness for a degree [3]. Moreover, students’ readiness for a career has been documented as being greater when they have one or more mentor/advisor [11].

PhD students highlighted the importance of good supervision when doing doctorates, and most reported positive comments about their supervisors [21]. In the study by Lee [25], students of a professional doctorate programme have been reported to welcome a supervisor with a different background, who could develop their knowledge and skills and add new dimensions to their research, while supervisors were more likely to want to match their background with that of the students. From the perspective of supervisors, critical thinking, independence and autonomy in the supervision process have been highlighted, whereas students wanted help and support to further develop their critical thinking and writing skills. Students see supervision as a mutual relationship with mutual learning. Also, they wanted to use their practical expertise together with doctoral studies in order to generate knowledge for application in practice, rather than to only learn how to apply other evidence [25]. Moreover, PhD students reported positive experiences regarding the timing of feedback received from their supervisors, but they reported that this feedback was not always helpful and was often accompanied by conflicting expectations [21]. In a study of PhD and DNP students, stressors which significantly predicted students’ leaving the programme were primarily related to the relationship with the faculty/advisor [54].

#### Process level (4) Doing doctorate abroad

Doing a doctorate abroad has been documented as promoting learning independence and increasing the understanding of cultural differences [47]. Moreover, in a qualitative study of 17 students representing nine different countries from six different UK universities, the majority (*n*=13) reported expecting greater focus on professional issues within their programme and were surprised at the almost exclusive emphasis on research. They expected to have more clinical training. Additionally, respondents highlighted the need to adapt to the self-directed autonomous nature of learning at a doctoral level, which was very difficult, as also reported previously [47].

Many foreign students noted that their educational backgrounds trained them to describe and replicate knowledge rather than create it. However, students have stated that it was good to find their own voice and articulate their own ideas [24]. Additionally, they indicated the enormous challenge of studying in English. When abroad, students described a strong need for support through supervisors, interactions with the department/institution and relationships with other students and wider social networks. Supervision has been reported to be different to that in students’ countries; they were supposed to be more independent in their work, and not told what to do, but supervisors were both approachable and friendly [24].

Struggling with loneliness, isolation and the cost of living in the UK have been also underlined. Most participants carried the weight of high expectations from their family, colleagues and sponsors on their shoulders, which, in some cases, clearly led to chronic anxiety about whether they would succeed [24].

#### Outcome levels (1) Academic performance outcomes in doctoral programs

Six common academic performance outcomes in nursing PhD programs have been identified to date: comprehensive examination (80 %), ongoing minimum graduate GPA (79 %) of 3.0, formal dissertation (82 %): chapter format (77 %); time to degree attainment (71 %); degree attainment (71 %); and time to candidacy (63 %) [49]. PhD programmes mostly end with a dissertation, while quality improvement projects and the translation of evidence-based practice have mostly been reported for DNPs [3].

With regards to the content of the thesis, Jensen [35] discovered that a few have been developed upon a nursing theory, whereas the majority of PhD graduates employed a traditional format for their dissertation, and over 40 % of them never published their dissertation findings in peer-reviewed journals [36]. In a study of 79 PhD programs in the US, 84 % offered the traditional format for dissertation and 71 % the manuscript option format, while 59 % offered both. The manuscript/publication format has been chosen as it may increase the transition of student nurse scholars to academic positions and provide preparation for the role. However, students with no academic writing skills are challenged; moreover, there is no agreement regarding the number of manuscripts and their status, and whether it should be already published, submitted or reviewed [37].

Regarding the doctorate duration, students who enter PhD programs as undergraduates have been reported to need less time cumulatively to finish a doctorate compared to those entering after a Master’s. However, on average, undergraduate students take longer to complete a PhD (from 5.2 to 5.9 years) in comparison to those who start a PhD after a Master’s education (from 5 to 5.1 years) [16, 30]. With regards to the DNP, the average length of time for students to finish the MSN-DNP programme was 2.43 years, whilst BSN-DNP was 3.8 years [50]. Time for degree completion was more important for DNP than PhD students; family and job obligations interfered with studying and writing [15].

#### Outcome levels (2) Doctoral graduates’ competences

Findings from PhD and DNP students and recent graduates have reported a lack of preparation for faculty roles, specifically for teaching [12], as also reported by Nehls et al. [16] in PhD students with different entry paths. Graduates with a PhD have become both a required and preferred option for teaching positions in many universities; given that teaching generally occupies the majority of the faculty’s time, they have been reported to be inadequately prepared according to the priority given to research [19].

Despite these issues, Fang et al. [30] reported that the majority of PhD students at the end of the programme plan their academic career in teaching more than in research due to the interest in teaching and the perceived contribution of research to patient care. Interestingly, nearly half of students surveyed who had non-academic career plans at the beginning of the programme changed their mind during the doctoral study. Similar findings were documented by Bai et al. [14], who found that 60.7 % of PhD students want to work in an educational institution, especially reputable universities. Their desired field of work was nursing education in the majority (75.3 %) and the clinical setting in only a few (16.9 %).

In contrast, PhD students who entered a doctoral programme via an early entry pathway, such as pre-baccalaureate or post-baccalaureate, have been reported to more often choose a research career [16]. However, they have also noted concerns regarding their clinical competences [55]. In this context, PhD education has been recommended to develop their clinical competences in order to prepare them to provide the clinical educator role.

Moreover, some DNP graduates have been reported to come back to the doctoral programme to do a PhD [56]. Their decision was mostly motivated by wanting to know more, especially regarding translating research into practice and implementing research findings, but also with the social tension of not having skills or competences to apply for specific roles at the faculty level.

#### Outcome levels (3) Doctoral students/graduates’ satisfaction

Doctoral education has been reported to be worth the time commitment and the money invested; PhD students and graduates, more than their DNP colleagues, have been documented to believe that doctoral programs prepared them extremely well for research activity and faculty roles [15].

#### Outcome levels (4) Doctoral graduates challenges

In a qualitative study among the PhD and DNP-prepared faculty on tenure track in academia [17], the importance of the development of meaningful partnerships and continuity was reported. Also, the need to balance responsibilities was documented, as was having time for research, publications, and the management of administrative requests. Both PhD and DNP staff on the tenure track have reported hostile treatment and criticism by senior faculty members; however, ‘degree shaming’ has been reported more often by DNP staff. Despite this, many staff on the tenure track have also reported being valued and appreciated. Additionally, they showed the need to be mentored, supported and coached, specifically in more advanced research.

In Nicholes and Dyer [24], 61.3 % of the DNP faculty were eligible for tenure. However, there is a concern that they are not trained well enough for the research activity, which may influence the development of the nursing discipline.

In the case of PhD nurses who are APNs working in a clinical environment, even though they undertake several important roles for nursing practice development (such as clinical inquiry, research skills and changing practice), they have encountered challenges with integrating themselves into the team without the support of nursing leaders [48]. According to Orton et al. [57], RNs with a PhD in the clinical environment also experience challenges: they have been reported to want to change practice, but in doing this, they must face barriers. They have admitted that their motivation to lead the evidence-base practice is strong, but doing their own research was difficult, mostly because of the lack of time. They have often been assigned to the clinical education of nursing students, or to assist colleagues in developing knowledge and skills. Therefore, further role clarity is needed in clinical settings. Similar findings have been reported by Sørensen et al. [54] who surveyed nurses with PhDs working in Nordic university hospitals. They reported sharing their work time between research, teaching, supervision and administration given that the majority of them held positions shared between the university and the hospital. Moreover, Andreassen and Christensen [38] underlined the fact that nurses with a PhD working in clinical areas are seen by their colleagues and by themselves as those who implement research results into practice. They are seen as a resource of health care institutions; however, their position in clinical settings is uncertain.

## Discussion

We have performed a Scoping Review with the aim of assessing the state of the art and issues regarding doctoral programs of nurses. A significant number of studies have emerged in the last 10 years, mainly in the US and Europe, with a few examples of international approaches [5, 45] suggesting an impulse in this direction. Moreover, studies are mainly quantitative and qualitative, where the experiences/expectations of students, doctorates and faculty members, as different groups (e.g. [3]) or integrated (e.g. [55]), have mainly been investigated. Therefore, the available evidence is mainly descriptive of different aspects of education, highlighting that more longitudinal or experimental [1] studies are needed in this field of research to investigate the effects of this education as well as its changes, in the long-term, from different points of view, including that of students, doctors and faculty members.

At the structural level of doctoral programs, a great variability of admission criteria has been documented to date, some regarding the academic preparation or potentialities (e.g., GPA, GRE, writing essay, [49]), and others regarding the attitudes and competences as either actual or potential, as certified with a letter of recommendation. An ample debate has also emerged regarding solutions concerning the minimum requirements of clinical hours for younger candidates (e.g. [44]), approaching doctoral studies in the short-term. Some data have already been produced regarding early undergraduate entry in a PhD programme on good research productivity, research career interests and longer time productivity for the nursing discipline [16]. However, the experiences available are mainly descriptive, and there are calls to conduct studies investigating predictors of doctoral programme success regarding all of these aspects with the intent to inform the best decisions regarding the establishment of a common framework of admission criteria that might be useful, especially for transnational doctoral programmes. On the side of candidates/students, issues associated with the financial implication both in the short- (fees) and long-term (e.g., the impact of future salary) should be considered in those countries where a limited number of doctoral-prepared nurses are available. Issues regarding the preparation of the faculty have been less well-investigated to date [13], while emphasis has been placed regarding the mission of doctoral education, not only concerning the differences between PhD and DNP programmes but also regarding their collaboration with the practice. The aspect of cooperation between graduates of these two programmes are also highlighted by position papers published by the American Association of Colleges of Nursing [22, 23].

With regards to process dimensions, a regular upgrading of the programme contents [10] as well as in the modalities of the final examination [53] are recommended, given that studies have reported some form of stability over the years [10], while these programmes are required to respond to emerging research priorities, innovations in methodologies and competence, as underlined by the American and Canadian organisations working for nursing science excellence [22, 59, 60]. Additionally, the majority of studies available have reported a difference in the perceptions of doctoral programme quality between the faculty and students, with high values among the first (e.g. [7, 8]): in a student-centred approach, understanding this gap and promoting improvements is recommended as a strategy to align the programme delivered to the expectations of attendees. Moreover, the quality of a doctoral programme is also enhanced by the research unit/centres and support offered to students – suggesting therefore that each doctorate programme should be equipped by the resources established at the academic level – thus allowing students to access not only traditional resources (faculty members, supervisors, and librarian) but also a centre devoted to supporting research. Special equipment should be ensured in those doctoral programmes hosting foreign students which have been documented to have additional needs requiring appropriate support (e.g. [24, 47]).

A good relationship of PhD students with their supervisors is important for programme completion, as well as for growing as a person and a scientist. To date, different dimensions have been debated in the supervisor’s preparation, background, number of students to supervise, and number of mentors for each student (e.g. [11, 25]), leaving the supervision process still under-researched [24]. Moreover, available studies seem to have considered singular elements of the process rather than the quality of the entire academic environment as being capable or not of promoting excellence in doctorate education. This is visible in the position statement launched by the American Association of Colleges of Nursing [22], as well as in the Quality Standards for Canadian Doctoral Education in Nursing, which underlines that the criteria required for excellence in PhD education include: active faculty researchers who would be capable of mentoring PhD students in research and helping with socialisation that is important in the competitive context; and opportunities for the active engagement of doctoral students in a scholarly environment with recognition of their contribution in the discipline development [59].

Finally, regarding outcomes, there has been no clear set of indicators established to date as doctoral programme outputs. A set of agreed outcomes measuring both the process (e.g., PhD/DNP duration) and end points (e.g., publications) in the short- and long-term (career achievements) in addition to the degree of satisfaction [15] are encouraged. All of these might support evaluation of the effectiveness of improvements – also at the international levels. Career plan expectations have been reported to change over the years and are different: in the clinical arena for DNP, and in teaching for PhD, as well as early entry [30]. The problems documented in the literature suggest that at least elective courses should be promoted to improve teaching and clinical competences. However, challenges have been underlined in the following career, not only due to the lack of some competences, but also due to entering a hostile academic environment [17] and in clinical practice where the support of leaders is crucial [48]. A wider career preparation should be an important element of doctoral programs from the initial stages [37]; also, in this case, more studies are required to understand factors promoting and hindering success in the transition from the student’s role to a doctorate position, both in the academic and clinical arenas, in order to design effective strategies. These strategies have also been underlined by the American Association of Colleges of Nursing and other stakeholders [22, 60]. Most doctoral students have reported the plan to work in educational institutions, but not many PhD graduates in clinical settings [14]; therefore, there is a need to prepare students for faculty roles without neglecting the clinical area [15].

In terms of roles, establishing the DNP has been recognised as a step forward in developing clinical nursing practice, as the number of nurses with a DNP is increasing rapidly. However, as numbers of PhD nurses decrease, it can be seen as a threat regarding the development of science in nursing as PhD candidates decrease, and they are seen as future scientific scholars [1, 58]. Moreover, degree confusion may be observed across the world considering that DNP is popular in only a few countries, mostly in the US [26].

This Scoping Review has several limitations. Firstly, only three databases were searched, with inclusion criteria limited to only publications in English; moreover, despite the systematic method used, some papers might have been missed. In addition, no grey literature has been searched, as more emphasis was placed on peer-reviewed primary studies published up to January 2020, thus missing publications which were more recent (e.g. [61]). Additionally, review/conceptual papers were also not included, so this is recommended for future analyses, as the discussion among scholars in this regard is lively e.g., [1, 4, 58]. When analysing the included studies, any differences have been introduced between PhD and DNP programmes that have been considered together according to the main aim of the study, while differences, if any, have been highlighted when reporting findings. Moreover, we used the Donabedian framework [43] to organise the study findings, given their ample variety. However, our study did not intend to assess the quality of doctoral programmes in their triad of structure, process and outcome dimensions.

## Conclusions

Doctoral education is expected to bring very complex outcomes – to prepare graduates as experts in the discipline, education, research, clinical practice, leadership, and policy-makers. Considering studies published in the last 10 years, deep discussion regarding doctoral programs for nurses is being provided by scholars and nursing leaders in the US and in Europe. Doctoral programs are different across countries, with no visible cooperation of scholars internationally; their structure and content has been reported as stable over the years, thus not following the research development in nursing, discipline and practice expectations.

Reflecting on the mission of the doctoral programmes, which will help to prepare future scientists equipped with strong competences in research methodology, there is a need to move the research produced from descriptive to more robust approaches that are capable of intercepting the effects of this education in their different features in the short- and long-term, in order to inform the establishment of evidence-based doctoral education pathways across the world.

Studies investigating predictors of success informing an evidence-based approach regarding the admission criteria, as well as regarding other process elements (e.g., the quality of the programme, the quality of the supervision) are recommended. Alongside the investigation of singular elements, scaling up the perspective by considering the quality of the entire academic environment as capable or not of promoting excellence in doctorate education is strongly suggested given the acknowledged importance of the environments on the learning process.

Doctorates in nursing should be equipped with appropriate resources and should embody an appropriate degree of continuing innovation in their programmes, as well as rigours evaluating outcomes in the short- and long-term. In this light, national and global strategies might be useful to ensure consistency and enhance the quality of the programmes and resources for doctoral education in nursing, as well as to link them to a university career plan. Government agencies should support the establishment of doctorate education; international professional organisations (e.g., the International Network for Doctoral Education in Nursing, International Council of Nurses; the Theta Tau International; European Academy of Nursing Science) might play a pivotal role by proposing doctorate frameworks [45]. It may be useful specifically for those countries which start with their doctoral programs in nursing or those who just plan to open it. However, career advancement, both with regards to clinical and academic roles, should be monitored in order to understand the implied factors and wastes in this context in order to prevent overeducated nurses not engaging in roles according to their competences.

## Data Availability

All data generated or analysed during this study are included in this published article.

## Abbreviations

APN: Advanced Nurse Practitioners
BSN: Bachelor degree
DNP: Doctor of Nursing Practice
GPA: Grade Point Averages
GRE: Graduate Record Examinations
MSN: Master’s degree
PhD: Doctor of Philosophy
PRISMA-ScR: Preferred Reporting Items for Systematic reviews and Meta-Analysis extension scoping reviews statement
